# P90 Targeting biofilm architecture and virulence regulation in *Staphylococcus aureus* skin infections using fruit-derived polyphenols

**DOI:** 10.1093/jacamr/dlaf230.097

**Published:** 2025-12-04

**Authors:** Bryan Moreno-Chamba, Julio Salazar-Bermeo, Sergio Navarro-Coves, María Concepción Martínez-Madrid, Domingo Saura, Manuel Valero, Victoria Lizama, Nuria Martí

**Affiliations:** Instituto Universitario de Ingeniería de Alimentos-FoodUPV, Universitat Politècnica de València, 46022, Valencia, Spain; Instituto de Investigación, Desarrollo e Innovación en Biotecnología Sanitaria de Elche (IDiBE), Edificio Torregaitán, Universidad Miguel Hernández de Elche (UMH), 03202, Alicante, Spain; Instituto Universitario de Ingeniería de Alimentos-FoodUPV, Universitat Politècnica de València, 46022, Valencia, Spain; Instituto de Investigación, Desarrollo e Innovación en Biotecnología Sanitaria de Elche (IDiBE), Edificio Torregaitán, Universidad Miguel Hernández de Elche (UMH), 03202, Alicante, Spain; Instituto Universitario de Ingeniería de Alimentos-FoodUPV, Universitat Politècnica de València, 46022, Valencia, Spain; Instituto de Investigación, Desarrollo e Innovación en Biotecnología Sanitaria de Elche (IDiBE), Edificio Torregaitán, Universidad Miguel Hernández de Elche (UMH), 03202, Alicante, Spain; Instituto de Investigación, Desarrollo e Innovación en Biotecnología Sanitaria de Elche (IDiBE), Edificio Torregaitán, Universidad Miguel Hernández de Elche (UMH), 03202, Alicante, Spain; Instituto de Investigación, Desarrollo e Innovación en Biotecnología Sanitaria de Elche (IDiBE), Edificio Torregaitán, Universidad Miguel Hernández de Elche (UMH), 03202, Alicante, Spain; Instituto Universitario de Ingeniería de Alimentos-FoodUPV, Universitat Politècnica de València, 46022, Valencia, Spain; Instituto de Investigación, Desarrollo e Innovación en Biotecnología Sanitaria de Elche (IDiBE), Edificio Torregaitán, Universidad Miguel Hernández de Elche (UMH), 03202, Alicante, Spain

## Abstract

**Background:**

Antimicrobial resistance (AMR) threatens global health, with *Staphylococcus aureus* remaining one of the most concerning pathogens due to biofilm formation, persistence on skin, and recurrent infections. A major driver of this persistence is biofilm formation, which protects bacterial cells from antibiotics and host defences, limiting the effectiveness of current therapies, especially since conventional antibiotics often fail to eradicate biofilms. Novel, sustainable strategies that attenuate virulence, disrupt biofilms and reduce the burden on skin host during infections, especially without fuelling AMR.

**Objectives:**

To determine the antibiofilm, anti-virulence and host-protective effects of polyphenolic extracts from grape (GBE), strawberry (SBE) and cherry (CBE) by-products as alternative candidates for reducing prevalence of *S. aureus* skin infections.

**Methods:**

After confirming the MIC of samples, the extracts were tested against *S. aureus* biofilms at early adhesion, pre-formed and mature stages. Biofilm biomass, exopolysaccharide (EPS) production and intracellular reactive oxygen species (ROS) were quantified. Quorum sensing (QS) interference was assessed using *Pseudomonas aeruginosa* autoinducers and swarming motility assays. Human keratinocytes (HaCaT) were used for cytotoxicity testing, bacterial adhesion assays and interleukin-6 (IL-6) measurement following *S. aureus* exposure.

**Results:**

Polyphenolic extracts inhibited biofilm formation by 50% at 2× to 1/4×MIC, especially initial biofilms, while only bactericidal concentrations affected matured biofilms. Samples also reduced EPS production by 50% and lowered intracellular ROS by 80% (all *P<*0.001). Extracts disrupted QS signalling autoinducers and decreased bacterial swarming motility. In keratinocyte infection models, adhesion of *S. aureus* was reduced by 50% with GBE and SBE, with improved effects than penicillin/streptomycin (*P<*0.001), while IL-6 secretion was suppressed by 60% compared with untreated controls (*P<*0.001). All extracts were non-cytotoxic at effective concentrations, when compared to untreated HaCaT cells (*P>*0.05).

**Conclusions:**

Fruit-derived polyphenols act as non-traditional anti-infective agents, disarming *S. aureus* by disrupting biofilms and virulence traits while also dampening host inflammatory responses. By targeting pathogenicity rather than viability, these compounds may reduce selective pressure for resistance and avoid collateral damage to the skin microbiome. Moreover, valorization of fruit-processing by-products provides a sustainable route for developing affordable adjuncts or alternatives to antibiotics. These findings support further development of plant-derived polyphenols as promising candidates in the global fight against AMR. However, further studies must be conducted to confirm this potential.

